# Usefulness of apical area index to predict left ventricular thrombus in patients with systolic dysfunction: a novel index from cardiac magnetic resonance

**DOI:** 10.1186/s12872-018-0988-9

**Published:** 2019-01-11

**Authors:** Yodying Kaolawanich, Thananya Boonyasirinant

**Affiliations:** Division of Cardiology, Department of Medicine, Siriraj Hospital, Mahidol University, 2 Wanglang Road, Bangkoknoi, Bangkok, 1070 Thailand

**Keywords:** Thrombus, Systolic dysfunction, Left ventricular geometry, Cardiac magnetic resonance, Apical area index

## Abstract

**Background:**

LV systolic dysfunction presents an elevated risk of thromboembolism. Previous studies demonstrated low left ventricular ejection fraction (LVEF), ischemic cardiomyopathy and increased myocardial scarring as independent risk factors for LV thrombus formation. Structural changes that alter the size and shape of LV apex may have a significant role in predicting LV thrombus, but there is no definite evidence exists in this entity.

**Methods:**

A case-control cardiac magnetic resonance (CMR) study of 150 patients with LV systolic dysfunction (LVEF < 40%; 30 patients with LV thrombus and 120 patients without thrombus) was performed. Factors associated with thrombus including sphericity index and ‘new’ apical area index (ratio of apical area to entire LV area from a cine four-chamber view) were evaluated.

**Results:**

Average age was 63.48 ± 12.82 years and mean LVEF was 29.22 ± 8.53%. Patients with LV thrombus had significantly higher apical area index than those without thrombus (46.5 ± 3.27 vs. 42.71 ± 3.02, *p* <  0.001) while sphericity index in both groups was not different (1.63 ± 0.27 vs. 1.67 ± 0.19, *p* = 0.57). Univariate analysis revealed that male gender, prior myocardial infarction, presence of apical aneurysm, ischemic-typed scar, apical scar and apical area index were associated with thrombus. Further, multivariate analysis showed only apical area index and apical scar as independent predictors for thrombus formation.

**Conclusion:**

Apical area index from CMR is a new index to predict LV thrombus in patients with LV systolic dysfunction and may have a future role in early anticoagulant therapy.

## Introduction

Patients with left ventricular (LV) systolic dysfunction are at increased risk of thromboembolic events that may result in major adverse cardiac sequelae. According to previous echocardiographic studies, the prevalence of LV thrombus in patients with LV systolic dysfunction was 8–19% [1, 2]. Thus, accurate detection of LV thrombus affects clinical outcomes and appropriate management.

Cardiac magnetic resonance (CMR) provides high-resolution images, good reproducibility and tissue characterization for detection of thrombus [3]. Various CMR sequences are dedicated for thrombus identification with excellent diagnostic accuracy, and sensitivity and specificity at 88 and 99%, respectively [4]. These include gradient-echo, steady-state free precession (SSFP), and late gadolinium enhancement (LGE).

A recent comparative study demonstrated that CMR yielded more than 3-fold higher diagnostic accuracy for detection of LV thrombus compared with transthoracic and transesophageal echocardiography which detected thrombus based on anatomical appearance rather than tissue characteristics [4].

Two CMR studies demonstrated independent predictors of LV thrombus such as worsening left ventricular ejection fraction (LVEF), ischemic etiology, prior anterior wall myocardial infarction, and increased myocardial scarring [5, 6]. Apart from these factors, LV shape and geometrical changes may be associated with thrombus formation. However, no definite geometrical index exists to accurately predict thrombus.

Previous echocardiographic studies reported that some parameters including sphericity index (long-to-short axis ratio in a four-chamber view) and apical conicity index (apical-to-short axis length ratio in a four-chamber view) affected LV remodeling after anterior wall myocardial infarction [7]. Nevertheless, these indexes were not studied for thrombus prediction.

From our hypothesis, apical remodeling may associate with thrombus formation. Previous indices involved particular length measurements without a comprehensive evaluation of LV apex, thus we would like to introduce ‘apical area index’ (ratio of apical area to entire LV in a cine four-chamber view) as a new predictor of LV thrombus. The primary objective of this study was to compare apical area index of patients with LV thrombus with no thrombus group. The secondary objective was to determine the predictors of LV thrombus in patients with systolic dysfunction.

## Methods

### Study population

This case-control, single center study was approved by the institutional ethics committee. A total of 150 patients with systolic dysfunction (30 consecutive cases with thrombus and 120 consecutive controls without thrombus) who underwent cine, adenosine stress, and LGE-CMR during the same period at Siriraj Hospital (Mahidol University, Bangkok, Thailand) between August 2014 and January 2016 were enrolled. Systolic dysfunction was defined as LVEF by cine-CMR below 40%. Patients were most commonly referred for CMR examination to assess myocardial ischemia, myocardial viability, or causes of cardiomyopathy. Patients exhibiting the following criteria were excluded: 1) inability to perform CMR due to a permanent pacemaker or implantable cardioverter defibrillator implantation, 2) incomplete CMR examination, 3) history of allergy to gadolinium, 4) pregnancy, and (5) history of claustrophobia.

On the day of the CMR procedure, a complete medical history including cardiac risk factors, current medications including antithrombotic and heart failure drugs, information regarding coronary angiography results, prior coronary revascularization, previous myocardial infarction, and thromboembolic events were collected to assess potential predictors of thrombus.

Additionally, regarding the excellent ability of LGE-CMR for the diagnosis of infarcted myocardium, patients were considered to have ischemic cardiomyopathy if there was transmural or subendocardial scar pattern from LGE-CMR. All other patients were classified as having nonischemic cardiomyopathy.

### CMR protocol

Cardiac structure, function and thrombus were assessed from two CMR techniques, cardiac function by a SSFP technique and LGE using a 1.5 Tesla Gyroscan NT Philips scanner (Philip Medical Systems, Best, The Netherlands). Functional study was performed by the acquisition of images by a SSFP technique in Short-axis images were acquired every 8 mm throughout the entire LV. Long-axis images were obtained in standard 2-, 3-, and four-chamber orientations. Parameters for cardiac function were as follows: repetition time/echo time/number of excitations = 3.7 ms/1.8 ms/ 2, 390 × 312 mm field of view, 256 × 240 matrix, 1.52 × 1.21 reconstruction pixel, 8 mm slice thickness, and 70 flip angles.

For LGE imaging, images were acquired 15 to 20 min after intravenous injection of 0.2 mmol/kg gadolinium contrast agent (Magnevist, Bayer Schering Pharma, Berlin, Germany) with the following scanning parameters; echo time 1.25 ms, repetition time 4.1 ms, 15-degree flip angle, 303 × 384 mm field of view, 240 × 256 matrix, in-plane resolution 1.26 × 1.5 mm, slice thickness 8 mm and 1.5 Sensitivity-Encoding (SENSE) factor.

Additionally, long inversion time sequence was applied for increasing the accuracy for detection of LV thrombus, as mentioned in previous study [5].

### CMR analysis

CMR indices of LV function, geometry, and scarring were measured to determine whether these parameters were related to the presence of thrombus. CMR images in short-axis view were classified as the basal, mid, or apical part of the left ventricle. Segmentation of each slice was performed according to the recommendation of American Heart Association [8]. Left ventricular volume and mass was calculated and indexed for the body surface area. LVEF was quantitatively assessed by using end-systolic and end-diastolic volume calculated from the multiple slice short axis images. Wall motion of each myocardial segment was recorded as presence or absence of abnormal wall motion. Wall motion of each myocardial segment was also recorded as 5-grade system as follows: 1 = normal, 2 = hypokinesia, 3 = akinesia, 4 = dyskinesia, and 5 = aneurysm. LGE images were analyzed by visual assessment. The transmural extent of LGE was graded as follows: 0 = no LGE, 1 = 1–25%, 2 = 26–50%, 3 = 51–75%, and 4 = 76–100% compared to the myocardial area in that segment. Total scar volume as a percentage of LV myocardium was defined as the sum of all transmural extent of infarction scores throughout LV wall thickness / 4 times total number of segments [9]. Transmural scar was defined as transmural extension of LGE more than 50% of myocardial thickness in each segment, subendocardial scar was defined as transmural extension of LGE less than 51% of myocardial thickness.

### LV thrombus assessment

**Fig. 1:**
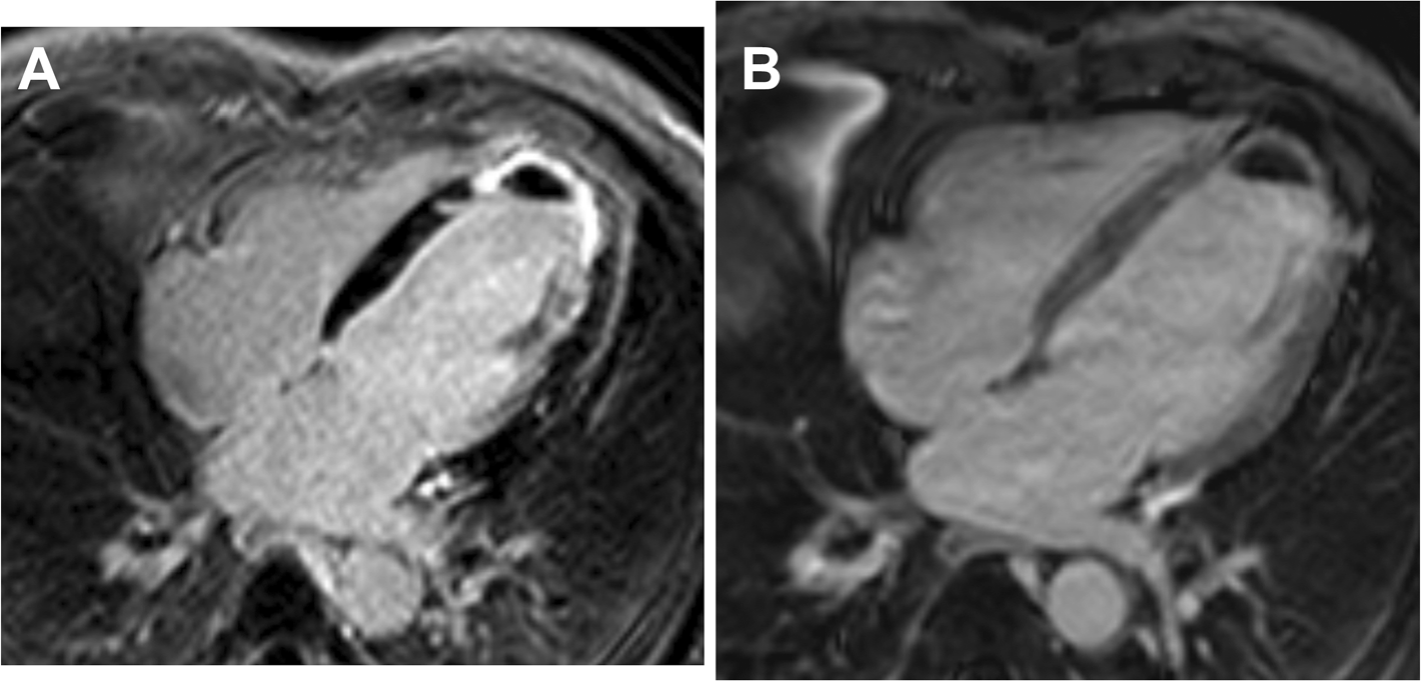
Presence of LV thrombus from LGE study. **a** LV thrombus from standard LGE. **b** LV thrombus from long inversion time sequence

Left ventricular thrombi detected by CMR on cine sequences were defined as filling defects within the LV cavity, typically adherent to regions of abnormal wall motion (hypokinesis, akinesis, or dyskinesis) then confirmed by LGE technique in Fig. 1a. In LGE images, thrombus was diagnosed as an LV mass with post-contrast inversion-recovery characteristics consistent with avascular tissue. Moreover, LGE-CMR can be further assessed for thrombus identification by increasing the inversion time (i.e., 600 msec) to selectively null avascular tissue such as thrombus. This ‘long inversion time’ approach provides an image that renders black thrombus and surrounding bright myocardium (Fig. 1b) [10].

### Geometrical parameters calculation

**Fig. 2:**
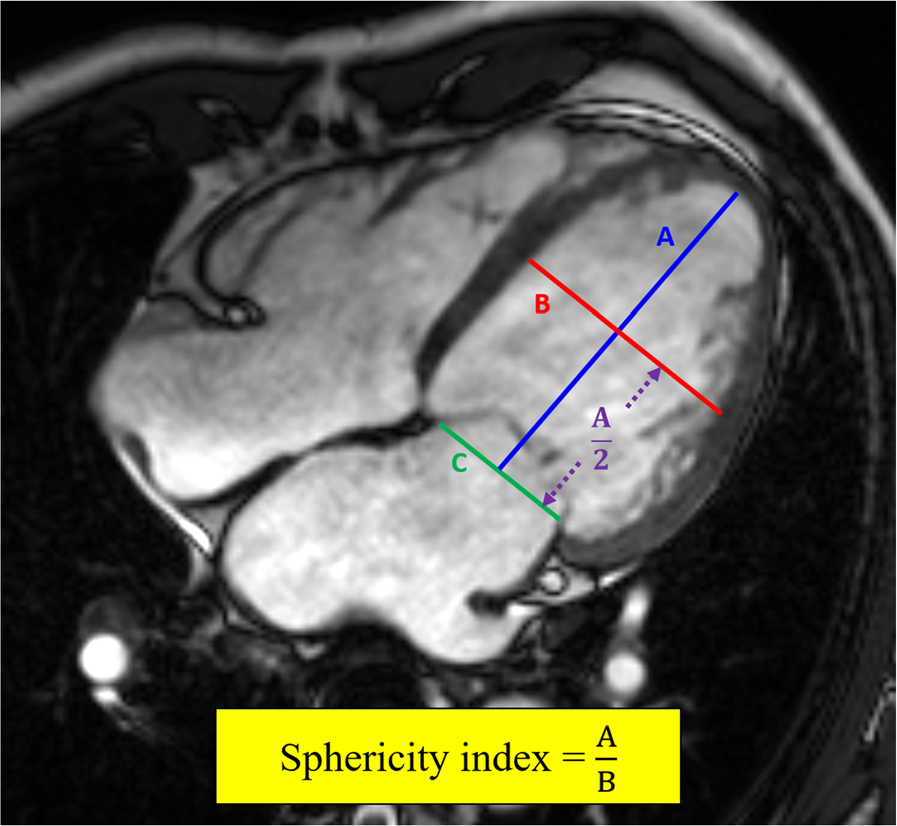
Calculation of sphericity index

Here, two indices were evaluated as sphericity index and ‘new’ apical area index. The sphericity index was described as the LV long-axis length divided by the mid LV diameter at end diastole for each in Fig. 2 [11, 12].

**Fig. 3:**
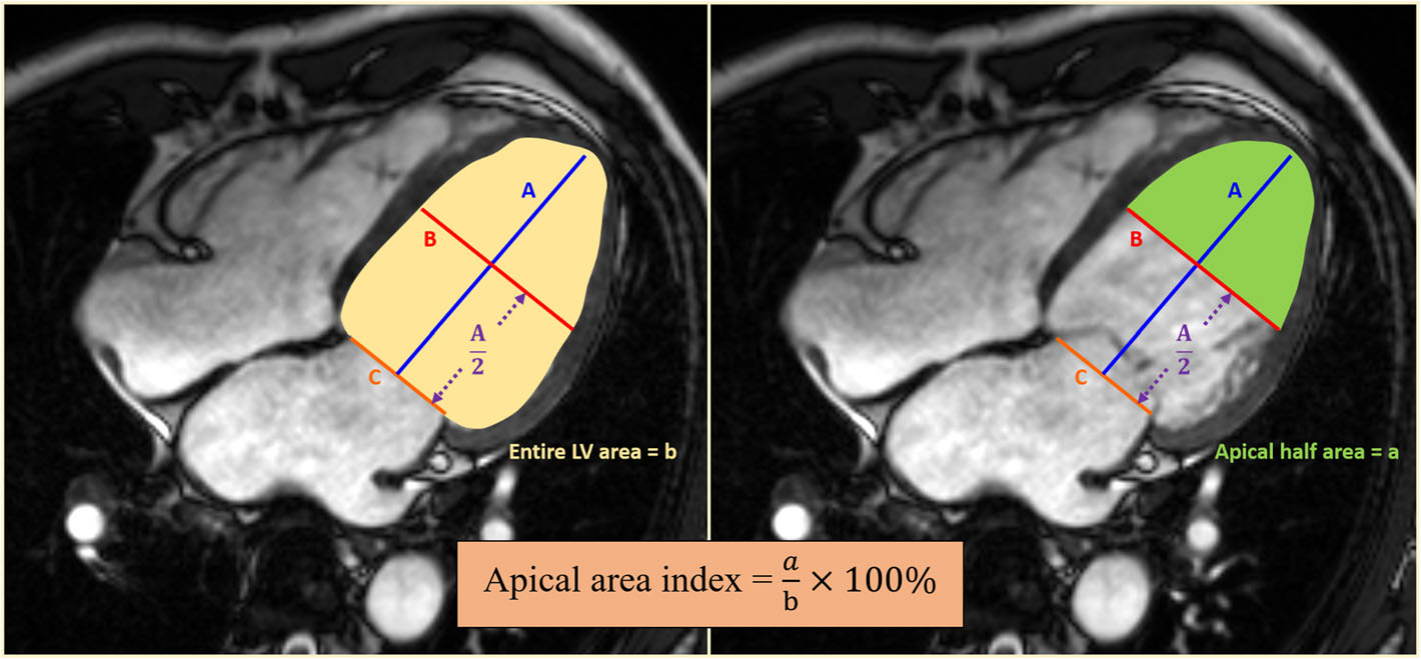
Calculation of apical area index

To represent the importance of apical remodeling, a ‘new’ apical area index was introduced to evaluate the predictor of thrombus. The apical area index was simply calculated as a percentage of an apical-half LV area (mid to apex) divided by an entire LV area from a cine four-chamber view in end diastole. Similar to the sphericity index, LV long-axis length was measured from the endocardial border of the mitral valve annulus plane to tip of apex, and the mid LV diameter was measured at the half-length from the LV long axis to tip of apex (Fig. 3).

### Intra- and inter-observer reliability

To assess intra- and inter-observer reliability of apical area index measurements by CMR, 20 patients were randomly selected to measure variability by the same observer 4 weeks after the initial analysis, and by the second independent observer blinded to the initial results.

### Statistical methods

Descriptive statistics, including frequency and percentage, were used for categorical variables. Continuous variables were reported as mean ± standard deviation for normally distributed variables and median (percentile 25 and percentile 75) for non-normally distributed variables. Normality of distribution of variables was examined by Kolmogorov-Smirnov test. Comparisons of categorical variables between patients with and without thrombus were performed using chi-square test or Fisher’s exact test. Continuous variables were compared using Student’s t-test or Mann-Whiney U test. Univariate and Multivariate analyses of thrombus predictions were evaluated using binary logistic regression analysis (Backward method) and presented as Odds ratio (OR) (95% confidence interval [CI]). Intra- and inter-observer reliability were analyzed by the intraclass correlation coefficient.

For all tests performed, a two-tailed *p*-value < 0.05 was statistically significant. PASW Statistic (SPSS) 18.0 (SPSS, Inc., Chicago, IL, USA) was used to perform all statistical analyses.

## Results

### Population characteristics

Clinical characteristics of the overall population are shown in Table 1. In total, 150 patients (103 male and 47 female) were consecutively enrolled with mean age of 63.84 ± 12.82 years. Almost a quarter of the subjects had prior myocardial infarction. Sixteen patients had history of a prior cerebrovascular event and 15.3% were receiving chronic warfarin therapy.

**Table 1:** Baseline characteristics

	Total (*n* = 150)	Thrombus (*n* = 30)	No thrombus (*n* = 120)	*p*-value
Age (years)	63.84 ± 12.82	60.93 ± 14.20	64.57 ± 12.41	0.17
Male gender	103 (68.7%)	26 (86.7%)	77 (64.2%)	0.02
BMI (kg/sqm)	24.27 ± 4.42	25.66 ± 5.50	23.92 ± 4.06	0.06
Atherosclerotic risk factors				
Hypertension	110 (73.3%)	26 (86.7%)	84 (70%)	0.07
Diabetes mellitus	49 (32.7%)	12 (40%)	37 (30.8%)	0.34
Dyslipidemia	108 (72%)	26 (86.7%)	82 (68.3%)	0.05
Smoking	22 (14.7%)	4 (13.3%)	18 (15%)	1.00
History of myocardial infarction	36 (24%)	13 (43.3%)	23 (19.2%)	0.006
Coronary revascularization (PCI)	9 (6%)	2 (6.7%)	7 (5.8%)	1.00
History of heart failure	88 (58.7%)	19 (63.3%)	69 (57.5%)	0.56
History of CVA or TIA	16 (10.7%)	4 (13.3%)	12 (10%)	0.53
Atrial fibrillation or atrial flutter	26 (17.3%)	3 (10%)	23 (19.2%)	0.24
Antithrombotic				
Aspirin	96 (64%)	21 (70%)	75 (62.5%)	0.44
Warfarin	23 (15.3%)	2 (6.7%)	21 (17.5%)	0.17
Heart failure medications				
Beta-blocker	113 (75.3%)	22 (73.3%)	91 (75.8%)	0.78
ACEI	64 (42.7%)	14 (46.7%)	50 (41.7%)	0.62
Angiotensin receptor blocker	32 (21.3%)	4 (13.3%)	28 (23.3%)	0.23
Spironolactone	23 (15.3%)	2 (6.7%)	21 (17.5%)	0.17
Furosemide	72 (48%)	13 (43.3%)	59 (49.2%)	0.57
Nitrate	40 (26.7%)	9 (30%)	31 (25.8%)	0.64
Digoxin	20 (13.3%)	3 (10%)	17 (14.2%)	0.77

*ACEI* = angiotensin converting enzyme inhibitors, *BMI* = body mass index, *CVA* = cerebrovascular accident, *PCI* = percutaneous coronary intervention, *TIA* = transient ischemic attack

Imaging parameters are demonstrated in Table 2. The cine-CMR showed evidence of advanced systolic dysfunction. Average LVEF was 29.22 ± 8.53% with apical left ventricular aneurysms presented in 22%. Extensive scarring was demonstrated from LGE images with mean scar volume 29.76 ± 15.72%. Transmural and subendocardial scar in the coronary vascular territory was defined as ischemic scarring pattern and found in 91 patients (60.7%). Apical scar was present in 47 patients (31.3%).

**Table 2:** CMR parameters

	Total	Thrombus	No thrombus	*p*-value
	(*n* = 150)	(*n* = 30)	(*n* = 120)	
LV function and morphology				
End-diastolic volume (ml)	238.87 ± 60.02	242.47 ± 55.91	236.71 ± 61.05	0.38
End-systolic volume (ml)	172.07 ± 57.75	181.90 ± 62.38	169.61 ± 56.54	0.30
Left ventricular ejection fraction (%)	29.22 ± 8.53	28.20 ± 10.38	29.48 ± 8.03	0.47
Left ventricular mass index (g/sqm)	76.16 ± 22.60	79.41 ± 22.58	75.35 ± 22.63	0.38
Presence of apical aneurysm	34 (22.7%)	15 (50%)	19 (15.8%)	< 0.0001
LV scarring				
Ischemic cardiomyopathy scar	91 (60.7%)	25 (83.3%)	66 (55%)	0.004
Scar volume (% LV)	29.76 ± 15.72	34.06 ± 14.69	27.97 ± 15.91	0.06
Presence of apical scar	47 (31.3%)	19 (63.3%)	28 (23.3%)	< 0.0001
Shape parameters				
Sphericity index	1.64 ± 0.21	1.63 ± 0.27	1.67 ± 0.19	0.57
Apical area index	43.46 ± 3.42	46.50 ± 3.27	42.71 ± 3.02	< 0.0001

CMR = cardiac magnetic resonance; LV = left ventricle/ ventricular

### Clinical and structural markers of the presence of thrombus

Thirty patients with LV thrombus from cine and LGE-CMR were mostly male and exhibited prior myocardial infarction without any differences in other coronary artery disease risk factors, atrial fibrillation, prior thromboembolic events, or baseline medication regimen including anticoagulation therapy.

Patients with LV thrombus did not differ from those without thrombus in aspects of LV volume and LVEF. Apical aneurysm was presented in significantly more patients with LV thrombus than without (50% vs. 15.8%, *p*-value < 0.0001). Significantly extensive apical scarring was found in patients with LV thrombus compared with no thrombus (63.3% vs 23.3%, *p*-value < 0.0001).

For LV geometrical indexes, patients with LV thrombus had significantly greater apical area index than those without thrombus (46.5 ± 3.27 vs 42.71 ± 3.02, *p*-value < 0.0001), while no difference of sphericity index was detected between patients with and without thrombus (1.63 ± 0.27 vs 1.67 ± 0.19, p-value = 0.57).

### Multivariate analysis

Six clinical and CMR variables including male gender, history of myocardial infarction, presence of apical aneurysm, ischemic cardiomyopathy scar pattern, apical scar, and apical area index were analyzed using multivariate analysis **(**Table 3**)**. Only the presence of apical scar and apical area index were determined as independent markers for thrombus.

**Table 3:** Univariate and multivariate analysis

	Univariate analysis		Multivariate analysis	
	OR (95% CI)	*p*-value	OR (95% CI)	*p*-value
Male gender	3.63 (1.19–11.09)	0.02	–	–
History of myocardial infarction	3.23 (1.37–7.57)	0.007	–	–
Presence of apical aneurysm	5.32 (2.23–12.66)	< 0.0001	–	–
Ischemic cardiomyopathy scar	4.09 (1.47–11.40)	0.007	–	–
Presence of apical scar	5.68 (2.42–13.34)	< 0.0001	3.56 (1.07–11.86)	0.04
Apical area index	1.49 (1.27–1.75)	< 0.0001	1.51 (1.23–1.84)	< 0.0001

### Intra- and inter-observer reliability

Moderate intra- and inter-observer reliability were shown for apical area index measurements by cine-CMR. In 20 patients, mean apical area index values were 44.05 ± 4.79 and 43.18 ± 4.13 (*r* = 0.73, *p* = 0.001) for the first observer in the initial analysis and 4 weeks later, respectively, and 42.52 ± 3.51 (*r* = 0.69, *p* = 0.001) for the second observer in the initial analysis.

## Discussion

Recent studies have shown independent risk factors for LV thrombus formation including low LVEF, ischemic cardiomyopathy, prior anterior wall myocardial infarction, and increased myocardial scarring [5, 6]. However, an established geometrical parameter for prediction of LV thrombus has remained unclear. Our study provides a new index for thrombus formation as ‘apical area index’. Patients with thrombus demonstrated significantly greater apical area index than without thrombus. Furthermore, our study demonstrated apical scarring as another strong independent predictor of LV thrombus.

### CMR evaluation of LV thrombus

Accurate detection of cardiac thrombus affects clinical outcomes and therapeutic management as LV thrombus provides a substrate for thromboembolic events and a rationale for anticoagulation. CMR enables LV thrombus to be detected based on intrinsic tissue characteristics related to avascular tissue composition. CMR tissue characterization for LV thrombus has been well-documented and validated compared to both pathological and clinical outcome reference standards.

LGE-CMR is widely used to differentiate between infarcted and viable myocardium based on relative differences in gadolinium-based contrast uptake. The technique can be used to identify thrombus and has been well validated in several different at-risk cohorts.

Long inversion time is another CMR sequence add-on standard LGE-CMR which increases the accuracy of thrombus detection, especially for mural thrombi which are difficult to identify by standard LGE imaging. Prolonging the inversion time produces an image that renders thrombus black with surrounding bright myocardium and accentuates the utility of CMR to evaluate patients with LV systolic dysfunction, as in our study.

Recent comparative studies have demonstrated that CMR yields superior detection of LV thrombus compared with echocardiography, which detects thrombus based on anatomical appearance rather than tissue characteristics. Among 160 patients undergoing LV reconstruction surgery (in whom pathology verification was uniformly available), Srichai et al. reported that CMR yielded more than 3-fold higher diagnostic accuracy for detection of thrombus then transthoracic echocardiography (87% vs. 27%) [4]. Improved accuracy was predominantly attributable to markedly higher sensitivity for CMR compared to echocardiography (88% vs. 23%).

Screening algorithms have been proposed that use the extent of wall-motion abnormalities on transthoracic echocardiography to trigger consideration of subsequent imaging with CMR [13]. High apical LV wall motion scores yield sensitivity approaching 100%, with specificity greater than 60%, and low apical LV wall motion scores allow appropriate avoidance of further testing in a substantial proportion of patients.

Previous evidence indicated CMR as a non-invasive modality of choice to diagnose LV thrombus. Moreover, our study emphasized the importance of apical remodeling to provide apical area index assessment.

### Markers of thrombus formation

From our study, patients with LV thrombus did not differ from those without thrombus in aspects of LV volume and systolic function, while male gender, history of myocardial infarction, apical aneurysm, apical scar, and ischemic scar were higher in the thrombus group. Most factors corresponded with prior studies such as history of myocardial infarction, ischemic scar and apical aneurysm [5, 14–17]. Apical aneurysm was found in 50% of patients with LV thrombus and was an important predictor of severe apical asynergy in our study.

We determined apical scarring as a new predictor of LV thrombus. Extensive apical scarring was found significantly more in patients with thrombus than without thrombus (63.3% vs 23.3%, *p*-value < 0.0001) as additional evidence demonstrating the significance value of apical change for thrombus formation.

Scar volume has previously been established as a risk factor for thrombus formation [5]. Our study found no difference of scar volume between patients with LV thrombus and those without. However, patients in both groups showed evidence of severe scarring (mean scar volume of all patients = 29.76 ± 15.72% LV) which was higher than previous studies. A trend of greater scar volume was presented in the thrombus group (34.06% vs 27.97%, *p* = 0.06).

### New apical index to fill the gap

Sphericity index and apical area index were the two geometrical parameters assessed for LV thrombus prediction in our study. The sphericity index was described according to substantial echocardiographic studies which evaluated LV systolic and diastolic dysfunction [11, 12]. One CMR study indicated that increased sphericity index was associated with reduced apical relaxation velocities as previously described [18]. Our hypothesis suggested that apical shape may be associated with LV thrombus formation. The sphericity index originates from the ratio of LV long axis to short axis in four-chamber view that may represent apical remodeling, while apical area index provides more information as it calculates the apex area ratio to entire LV, not only from dimensional evaluation.

From our results, sphericity index failed to demonstrate any difference between the two groups, while apical area index in the LV thrombus group was significantly higher than the group without thrombus. Apical area index measurement is simple with acceptable reproducibility and add-on information to predict LV thrombus in patients with systolic dysfunction.

Moreover, from multivariate analysis, only apical scar and apical area index were determined as independent predictors of LV thrombus. Combinations of these two factors will provide additional value in this aspect. This new index in other cardiomyopathies involving apex warrants further study.

### Study limitations

There were some study limitations, First, our study design cannot demonstrate prevalence of LV thrombus in the population; however, this was evident in previous study [5]. Second, a case-control study can only show an association between a predictor and an outcome without guaranteeing a causal relationship. Third, too few patients had undergone coronary angiography to provide a definite diagnosis of ischemic cardiomyopathy. Nevertheless, this study focused mainly on CMR parameters and the accuracy of LGE technique to differentiate these conditions was excellent. Forth, our study was a small single center study to initially prove the hypothesis. Further study which includes more patients or multicenter study is necessary for the validation of the results.

## Conclusion

Apical area index is a new simple predictor of LV thrombus formation from cine-CMR in patients with systolic dysfunction.

## Clinical application and future research

Results will be of value for two future research areas as 1) to identify the cut-off level of apical area index to accurately predicted thrombus in larger populations, and 2) to evaluate the association between apical area index and hard clinical outcomes such as stroke. If an association is present, patients may get benefits from early coagulation therapy. This entity warrants further study.

## Abbreviations

ACEI: Angiotensin converting enzyme inhibitors
BMI: Body mass index
CMR: Cardiac magnetic resonance
CVA: Cerebrovascular accident
LGE: Late gadolinium enhancement
LV: Left ventricular / ventricle
LVEF: Left ventricular ejection fraction
PCI: Percutaneous coronary intervention
SSFP: Steady state free precession
TIA: Transient ischemic attack
